# Chronic Effects of Static Stretching Exercises on Skeletal Muscle Hypertrophy in Healthy Individuals: A Systematic Review and Multilevel Meta-Analysis

**DOI:** 10.1186/s40798-024-00772-y

**Published:** 2024-09-28

**Authors:** Fabian Arntz, Adrian Markov, Brad J. Schoenfeld, Martin Behrens, David G. Behm, Olaf Prieske, Yassine Negra, Helmi Chaabene

**Affiliations:** 1 Department of Social- and Preventive Medicine, Research Focus Cognition Sciences, University of Potsdam, Am Neuen Palais 10, Building 12, 14469 Potsdam, Germany; 2 Faculty of Human Sciences, Division of Training and Movement Sciences, Research Focus Cognition Sciences, University of Potsdam, 14469 Potsdam, Germany; 3 Department of Exercise Science and Recreation, CUNY Lehman College, Bronx, NY USA; 4 Division of Research Methods and Analysis in Sports Science, University of Applied Sciences for Sport and Management Potsdam, Olympischer Weg 7, 14471 Potsdam, Germany; 5 School of Human Kinetics and Recreation, Memorial University of Newfoundland, St. John’s, NL A1C 5S7 Canada; 6 Division of Exercise and Movement, University of Applied Sciences for Sport and Management Potsdam, Olympischer Weg 7, 14471 Potsdam, Germany; 7 Higher Institute of Sport and Physical Education of Ksar Saïd, University of “La Manouba”, Manouba, Tunisia; 8 Research Laboratory (LR23JS01) «Sport Performance, Health and Society», Tunis, Tunisia; 9 Department of Sport Science, Chair for Health and Physical Activity, Otto-Von-Guericke University Magdeburg, Magdeburg, Germany; 10 Institut Supérieur de Sport et de l’Education Physique du Kef, Université de Jandouba, 7100 Le Kef, Tunisia

## Abstract

**Background:**

The chronic effect of static stretching (SS) on muscle hypertrophy is still unclear. This study aimed to examine the chronic effects of SS exercises on skeletal muscle hypertrophy in healthy individuals.

**Methods:**

A systematic literature search was conducted in the PubMed, Web of Science, Cochrane Library, and SPORTDiscus databases up to July 2023. Included studies examined chronic effects of SS exercise compared to an active/passive control group or the contralateral leg (i.e., utilizing between- or within-study designs, respectively) and assessed at least one outcome of skeletal muscle hypertrophy in healthy individuals with no age restriction.

**Results:**

Twenty-five studies met the inclusion criteria. Overall, findings indicated an unclear effect of chronic SS exercises on skeletal muscle hypertrophy with a trivial point estimate (standardised mean difference [SMD] = 0.118 [95% prediction interval [95% PI] = − 0.233 to 0.469; *p* = 0.017]) and low heterogeneity (I^2^ = 24%). Subgroup analyses revealed that trained individuals (β = 0.424; 95% PI = 0.095 to 0.753) displayed larger effects compared to recreationally trained (β = 0.115; 95% PI = − 0.195 to 0.425) and sedentary individuals (β = − 0.081; 95% PI = − 0.399 to 0.236). Subanalysis suggested the potential for greater skeletal muscle hypertrophy in samples with higher percentages of females (β = 0.003, [95% confidence interval [95% CI] = − 0.000 to 0.005]). However, the practical significance of this finding is questionable. Furthermore, a greater variety of stretching exercises elicited larger increases in muscle hypertrophy (β = 0.069, [95% CI = 0.041 to 0.097]). Longer durations of single stretching exercises (β = 0.006, [95% CI = 0.002 to 0.010]), time under stretching per session (β = 0.006, [95% CI = 0.003 to 0.009]), per week (β = 0.001, [95% CI = 0.000 to 0.001]) and in total (β = 0.008, [95% CI = 0.003 to 0.013]) induced larger muscle hypertrophy. Regarding joint range of motion, there was a clear positive effect with a moderate point estimate (β = 0.698; 95% PI = 0.147 to 1.249; *p* < 0.001) and moderate heterogeneity (I^2^ = 43%). Moreover, findings indicated no significant association between the gains in joint range of motion and the increase in muscle hypertrophy (β = 0.036, [95% CI = − 0.123 to 0.196]; *p* = 0.638).

**Conclusions:**

This study revealed an overall unclear chronic effect of SS on skeletal muscle hypertrophy, although interpretation across the range of PI suggests a potential modest beneficial effect. Subgroup analysis indicated larger stretching-induced muscle gains in trained individuals, a more varied selection of SS exercises, longer mean duration of single stretching exercise, increased time under SS per session, week, and in total, and possibly in samples with a higher proportion of females. From a practical perspective, it appears that SS exercises may not be highly effective in promoting skeletal muscle hypertrophy unless a higher duration of training is utilized.

*PROSPERO registration number*: CRD42022331762.

## Key points

- Chronic static stretching exercises result in an overall unclear effect on skeletal muscle hypertrophy. However, the range of the prediction interval reveals that the chance of a positive effect is greater than that of a negative effect, suggesting a potential hypertrophic benefit of static stretching.
- Subgroup analysis indicated that individuals with greater training experience achieved larger muscle gains compared to less-trained individuals following chronic static stretching.
- Meta-regression analyses suggested that a more varied selection of static stretching exercises, longer mean duration of single stretching exercise, and increased time under static stretching per session, week, and in total are associated with greater skeletal muscle hypertrophy.

## Background

Static stretching (SS) is frequently used in athletic, fitness, and clinical settings to increase joint range of motion (ROM) [1, 2]. Additionally, SS aims to mitigate injury incidence [3–5] and improve athletic performance [5–7]. Despite some studies showing that prolonged SS can acutely impair muscle strength and power, particularly when the total duration of the exercise per muscle group exceeds 60 s [4, 8], a recent systematic review with meta-analysis of 41 controlled trials indicated that chronic SS has the potential to improve muscle strength and power in healthy individuals [9]. This result was reinforced by another recent meta-analysis, which reported a small positive effect of long-term SS training on muscle strength in healthy individuals [10]. However, the specific mechanisms underpinning the observed increases in muscle strength and power following SS exercises have yet to be fully identified. In a narrative review focusing primarily on animal studies, Warneke et al. [11] suggested that there is insufficient clarity on the role of mechanical tension, hypoxia, fascial tissue, and neuronal mechanisms on stretch-mediated increases in human muscle strength and size. Interestingly, emerging evidence suggests that muscle hypertrophy may play a significant role in driving strength improvements after chronic SS [12–14].

Skeletal muscle hypertrophy is proposed to be a key determinant of muscle strength [15–18]. While the effect of resistance training on skeletal muscle hypertrophy is well-established [15, 19–22], the impact of SS remains uncertain [23]. The foundation of the concept of stretching-induced muscle hypertrophy can be traced back to studies using animal and in vitro models [24–27]. From an acute perspective, animal studies suggest that SS could activate mechanisms involved in muscle protein synthesis, including insulin-like growth factor [28, 29], hepatocyte growth factor (responsible for activation of satellite cells) [24], and the mammalian target of rapamycin (mTOR) pathway [30–32]. Moreover, in vivo studies in rats indicate that muscle stretching triggers the release of hepatocyte growth factor and activates satellite cells [33, 34].

Studies addressing the chronic effects of SS on skeletal muscle hypertrophy in humans show considerable heterogeneity, making it challenging to derive a comprehensive understanding of the impact of SS training on muscle hypertrophy. For example, while Panidi et al. [12] revealed a significant effect of 12 weeks SS on gastrocnemius cross-sectional area (CSA) in adolescent female volleyball players, e Lima et al. [35] reported no effects of 8 weeks SS training on biceps femoris architecture and muscle thickness of the vastus lateralis in healthy, physically active males. In a recently published descriptive review of the literature, Nunes et al. [23] concluded that passive low-intensity stretching does not seem to contribute to muscle hypertrophy and changes in muscle architecture. However, the authors also noted that when stretching is carried out with a certain level of tensile strain, such as when loaded or added between strength sets, it might have the potential to trigger muscle hypertrophy. This speculation was based on limited evidence and warrants further confirmation. It is worth noting that among the ten studies included in their review [23], five integrated stretching into resistance training programmes. As a result, the pure chronic effect of SS exercises on muscle hypertrophy cannot be distinguished from that of resistance training.

A recent meta-analysis investigated the effects of chronic SS training on muscle architecture in healthy individuals [36]. The results of the aggregated data from 19 studies indicated trivial to small positive effects on fascicle length at rest and during stretching with no effects on muscle thickness. However, besides excluding studies that included the contralateral leg as a control, the authors neglected to report the prediction intervals (PI), which means that the results may have been misleadingly interpreted. In fact, the PI is a very practical way to consider between-study heterogeneity (i.e., the extent to which true effect sizes differ between studies), which can be caused by factors such as including different participant groups or using different exercise modes [37, 38]. The PI can be described as the range in which the effect size of a novel study would likely lie provided that the study was randomly chosen from the same population as those in the studies considered in the meta-analysis [37, 39]. Of note, the advantage of PI in contrast to I^2^, for instance, is that they display heterogeneity in the same metric as the original effect size [37]. In addition, while it is encouraging to report some indicators of between-study heterogeneity such as I^2^ and Cochran’s Q, these are less practical tools to interpret between-study heterogeneity compared with PIs [39–41]. Moreover, there is evidence that the overreliance on I^2^ as an indicator of between-study heterogeneity may lead to misleading interpretations of the results [42, 43]. On the other hand, confidence intervals (CI) serve to depict the level of uncertainty surrounding the point estimate (or the range of potential effects consistent with the data) [44]. When between-study heterogeneity exists, the width of PI tends to be broader compared to CI. For instance, a CI might show a significant benefit for adopting a treatment (intervention) by not including zero, while the PI might include zero, indicating that the effect could vary between being negative or positive in future studies. Consequently, study conclusions might diverge if derived from the PI rather than the CI. It is worth mentioning that the sole reporting of CI offers inadequate insight into the underlying between-study heterogeneity [37]. This limitation could potentially lead to misleading interpretations of the findings. In this sense, the omission of reporting PI might imply the utilization and recommendation of treatments with an insufficient evidence base or the potential for harm in practical applications [38].

Generally, despite their relevance, PI are overlooked in meta-analytical studies [38, 45]. Hence, the primary objective of this study was to undertake a systematic review and meta-analysis on the chronic effect of SS exercise on muscle hypertrophy. This study stands out from the recently published [36] due to its unique approach in relying on the PI during the interpretation of the findings. Another distinctive facet of this meta-analysis is the inclusion of studies that not only employed a separate control group but also those that utilized the contralateral leg as control, allowing a more comprehensive overview of the existing literature. A secondary objective was to meta-analyse the effect of SS on joint range of motion (ROM). We also sought to elucidate the key SS training variables that may significantly influence skeletal muscle hypertrophy and joint ROM, aiming to facilitate the design of effective SS training prescriptions. Furthermore, we explored whether any increase in joint ROM resulting from SS training is associated with improved muscle hypertrophy. To the best of our knowledge, this specific relationship has not been previously investigated and provides important perspectives for understanding the broader implications of SS training.

## Methods

This systematic review with meta-analysis was registered in PROSPERO (CRD42022331762) and conducted in compliance with the Preferred Reporting Items for Systematic Review and Meta-analyses (PRISMA) statements [46].

### Search Strategy

The literature search was conducted independently and separately by two of the authors (FA and AM) in the electronic databases PubMed, SPORTDiscus, Web of Science, and Cochrane Library databases up to the 23rd of July 2023. The search was performed using a Boolean search strategy (operators “AND” and “OR”) and a combination of the following keywords:

(“Range of Motion” OR “Joint Range of Motion” OR “Joint Flexibility” OR “Passive Range of Motion” OR “Muscle Stretching Exercises” OR “Active Stretching” OR “Passive Stretching” OR “Static Stretching” OR “Dynamic Stretching” OR “Ballistic Stretching” OR “Isometric Stretching” OR “Proprioceptive Neuromuscular Facilitation” OR “PNF Stretching Exercise”) AND (“Hypertrophy” OR “Muscle Architecture” OR “Cross Sectional Area” OR “Muscle Volume” OR “Muscle Circumference” OR “Fascicle Length” OR “Muscle Power” OR “Explosive Strength” OR “Power” OR “Muscle Strength” OR “Strength”) AND (“Adolescent” OR “Child” OR “Adult” OR “Young Adult” OR “Older Adults” OR “aged” OR “seniors” OR “elderly”) AND (“controlled trial” OR “randomized controlled trial”). These keywords were identified using literature searches, expert opinion, and a controlled vocabulary (e.g., Medical Subject Headings [MeSH]). In addition, all included studies and corresponding meta-analyses were searched in so-called “snowball” searches [47] for further eligible publications. Only peer-reviewed publications written in English were considered for inclusion.

### Inclusion and Exclusion Criteria

Inclusion criteria for eligible studies were defined according to the PICOS (Population, Intervention, Comparison, Outcome, Study Design) approach [48]. The following criteria were defined: (1) Population: healthy participants without restriction regarding age, sex, or training status [49], (2) Intervention: SS interventions with a minimum duration of three weeks [36], (3) Comparison: active/passive control group/leg, (4) Outcome: at least one measure of muscle hypertrophy (i.e., muscle thickness, muscle cross-sectional area) or architecture (i.e., fascicle length) in a stretched muscle group, and (5) study design: (randomized) control trials with measurements at baseline and after completion of the intervention (within and/or between subjects). Studies were excluded if they included participants with existing medical conditions (e.g., musculoskeletal disorder, cardiovascular diseases), if there was no active/passive control group, if muscle hypertrophy/architecture was not assessed in the stretched muscle group, and/or if baseline or follow-up data were not available.

### Data Extraction

Data were extracted from included studies using a standardized Microsoft Excel template (FA) and cross-checked (AM). In case of disagreement regarding the extracted information or study inclusion, HC was consulted for clarification. Of note, measures of muscle hypertrophy before, after, as well during the intervention periods were considered. From the studies assessing the effects of SS on muscle hypertrophy, measures of joint ROM were also extracted and analysed. If single studies reported multiple measures of hypertrophy, all variables were included. If data were not reported in a way that allowed for the calculation of effect sizes, we contacted the respective authors to request appropriate data (i.e., mean ± standard deviation, raw data). If the authors did not respond to our request, we extracted relevant data from graphs where possible using the WebPlotDigitizer online software (v4.5, Ankit Rohatgi; https://apps.automeris.io/wpd/) [50].

From all included studies, the following information was extracted (a) lead author and year of publication; (b) comparator (i.e., within/between); (c) type of SS training (i.e., active/passive), (d) participants’ training status (i.e., tier 0–5) [49]; (e) percentage of females in the sample; (f) mean age of participants; (g) type of control condition (i.e., passive/active); (h) mean duration of single SS exercise; (i) number of sets per session; (j) number of different SS exercises; (k) weekly session frequency; (l) intervention period; (m) stretching intensity (i.e., below the point of discomfort [no pain]; at the point of discomfort [moderate pain]; above the point of discomfort [severe pain]), (n) assessed muscle group, (o) assessment method, and (p) measure of muscle hypertrophy or architecture as well as joint ROM. The extracted data was conducted by FA and cross-checked by AM.

### Methodological Quality of the Included Studies

The methodological quality of eligible studies was evaluated using the Physiotherapy Evidence Database (PEDro) scale [51]. The scale’s reliability, validity, and agreement with the Cochrane risk of bias tool are well established [51, 52]. Since blinding of participants, therapists, and assessors is difficult to employ in supervised SS interventions and thus is rarely implemented, items 5–7 were removed per recent systematic reviews [9, 53, 54]. Further, item 3 (i.e., “allocation was concealed”) was removed for studies using within-subject design interventions, as each participant receives the intervention as well as the control treatment. Accordingly, methodological quality was judged based on the percent of satisfied items (PEDro percent), to allow comparability of studies using within- and between-subject designs [9]. Data was then analysed using meta-regression statistics to assess possible moderating effects of study quality [55]. Additionally, overall and outcome-specific funnel plots [56], as well as graphical display of study heterogeneity (GOSH) plots [57] were used to depict publication bias and heterogeneity.

### Synthesis and Analyses

Meta-analyses and data processing were performed using the *‘metafor’* [58] and *‘tidyverse’* [59] packages in R (v 4.1.2; R Core Team, https://www.r-project.org/, Vienna, Austria). All analyses are available in the supplementary documentation (https://osf.io/snzba/). To assess effect sizes, we calculated the standardised mean change scores within SS and control conditions using baseline and post-test means and pre-test standard deviations. The standardised mean difference was calculated by subtracting control standardised mean change from SS standardised mean change and the corresponding variance was calculated as the sum of variances from both conditions [60]. The effect size’s magnitude was interpreted following Cohen’s thresholds [61]: trivial (< 0.2), small (0.2 to < 0.5), moderate (0.5 to < 0.8), and large (≥ 0.8).

Multilevel mixed-effects meta-analyses were used to calculate the effect size using individual studies as well as intra-study groups as random effects. Further, cluster robust point estimates were calculated using 95% CI and weighted by inverse sampling variance to account for within- and between-study variance. In addition, we calculated 95% PI to account for the uncertainty of the effects expected in future, similar studies [37, 40, 62]. Restricted maximal likelihood estimation was applied in all models. The potential effects of subgroups and regression variables were assessed using log-likelihood ratio test. The log-likelihood ratio test assesses whether adding additional variables to a model significantly improves the model’s fit to the data [63]. In this meta-analysis, if including a variable in the model improved the model’s fit, the subgroup effect was reported. Subgroup comparisons and meta-regressions were calculated for categorical (i.e., participant training status, type of SS exercise, stretching intensity, comparator, control condition, and assessed muscle group) and continuous (i.e., percent of females in sample, mean age, time under SS per exercise, time under SS per session, weekly time under SS, total time under SS, sets per SS exercise, sets per session, total number of sets, number of different SS exercises, weekly session frequency, and intervention period) variables, respectively. To explore the potential relationship between increased joint ROM and muscle hypertrophy, the change in joint ROM was integrated as a continuous subgroup variable.

To avoid dichotomization in our analysis, we reported p-values but did not employ traditional significance testing [64–66] and focused on the lower to upper limits of the PIs. As a secondary source of evidence, we consulted *p*-values. I^2^ statistics were applied [67], with I^2^ statistics being calculated for the overall, as well as within and between studies [68]. Heterogeneity was classified by I^2^ values as follows: low (I^2^ < 25%), moderate (25% ≤ I^2^ < 50%), high (50% ≤ I^2^ < 75%), or considerably high (I^2^ ≥ 75%) [67]. Of note, since pre-post correlations are rarely reported for within- and between-participant effects, we adopted a range of correlation coefficients (r = 0.5, 0.7, and 0.9) to examine the sensitivity of the results to these values. As the results were relatively insensitive to this range, we reported the results for r = 0.7.

## Results

### Study Characteristics

**Fig. 1:**
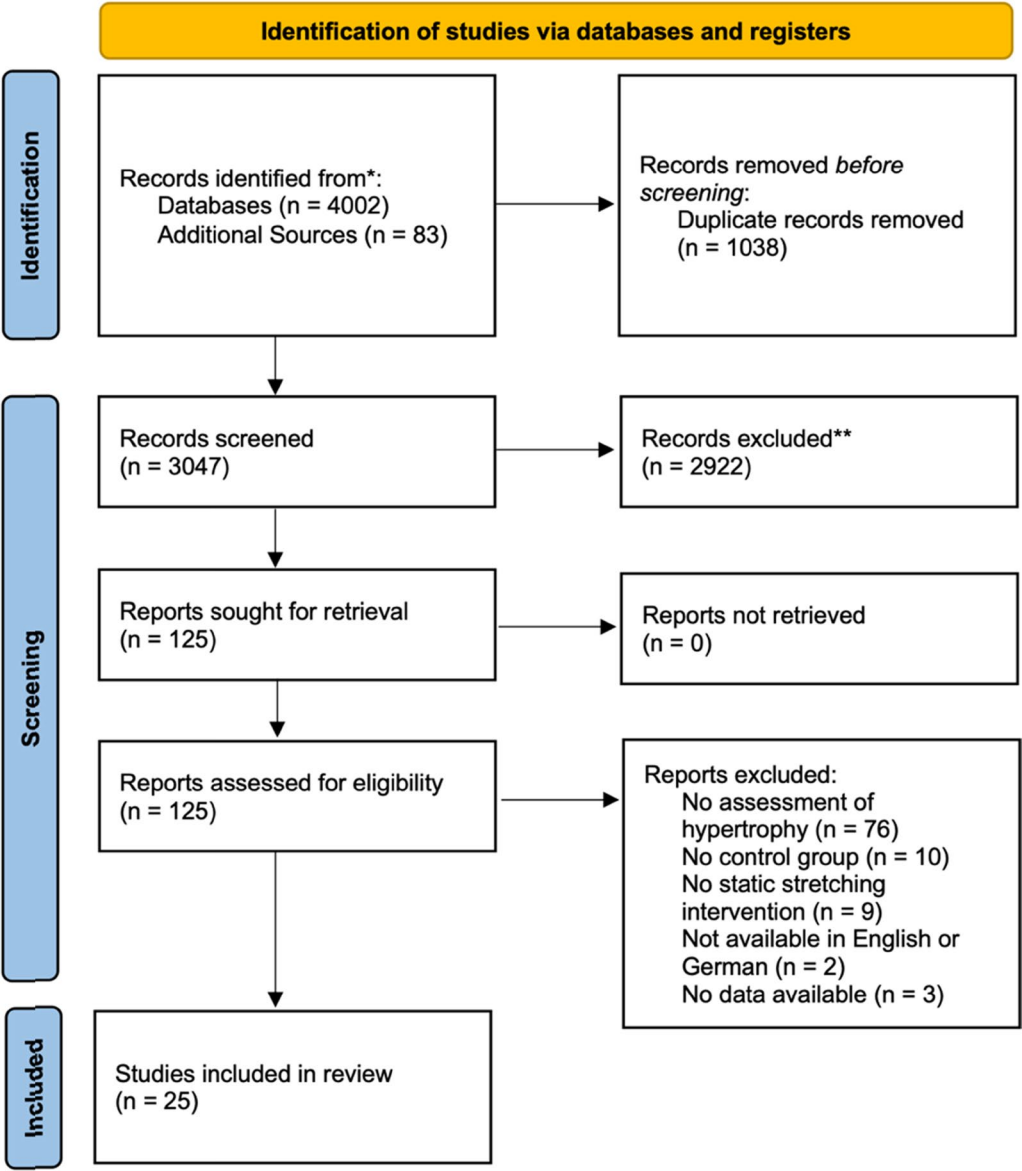
Flow chart illustrating the different stages of search and study selection

The literature search identified 4002 studies and snowball searches added 83. After removing duplicates and screening titles, abstracts, and full texts, a total of 25 studies met eligibility for inclusion. A PRIMSA flowchart of the search and review of studies is presented in Fig. 1. Overall, 710 (median per study = 23, range = 9 to 58) subjects participated across all included studies. Regarding the participants’ training status, two studies included sedentary participants, 15 included recreationally trained participants, three included trained individuals and six studies did not provide sufficient information to allow for classification. Six studies employed active SS, 18 studies employed passive SS and one study employed a mix of static and active SS. Seven studies had participants perform SS below the point of discomfort (i.e., no pain), eight studies had participants perform SS at the point of discomfort (i.e., moderate pain), 11 studies had participants perform SS above the point of discomfort (i.e., severe pain), and one study did not provide sufficient information to allow classification about discomfort. Regarding the comparator, 14 studies used a between-subject design, and eleven used a within-subject design. Four studies included an active control group while 21 utilised passive controls. Regarding the target muscle group(s) investigated, four studies assessed hypertrophy in the hip extensors (i.e., gluteus maximus), four evaluated knee extensors (i.e., quadriceps), six assessed muscle hypertrophy in knee flexors (i.e., hamstrings), and twelve assessed hypertrophy in the plantar flexors (e.g., triceps surae, gastrocnemius). Regarding the assessment method, b-mode ultrasound was consistently used by all included studies. Regarding participants’ sex, two studies included females, 15 included males, eleven included mixed groups, and one did not provide this information. The median of the mean age was 21.6 years (range = 13.5 to 28.2), the median of the mean duration of a single stretching exercise was 60 s (range = 20 to 3600 s), the median of the mean number of different stretching exercises was 1 (range = 1 to 6), the median of the mean number of sets was 3 (range = 1 to 8), the median weekly session frequency was 4 (range = 1 to 14), and the median intervention period was 6 weeks (range = 3 to 24).

Regarding study quality, studies using a within-subject design had PEDro scores ranging from 3 to 5 (median = 4.5) and studies using a between-subject design had PEDro scores ranging from 1 to 6 (median = 4.5). PEDro percent ranged from 14.3% to 85.7% with a median score of 69%. Full details of the included studies can be seen in Tables 1 and 2.

**Table 1:** Detailed characteristics of the included studies

Study	Study details^a^	Participants ‘ details^b^	Intervention details^c^	Pedro score (%)
Akagi and Takahashi [85]	Within-subject; passive; 19	Recreationally trained; 0; 23.7	Passive; 2-3-1-6-5; no pain	5 (83.3)
Andrade et al. [86]	Between-subject; passive; 58(41|17)	Recreationally trained; 46; 21.0	Passive; 1-52-5-12; severe pain	6 (85.7)
Blazevich et al. [87]	Between-subject; passive; 23(14|9)	NA; 0; 18.6	Passive; 0-3-1-14-3; moderate pain	5 (71.4)
Brusco et al. [88]	Within-subject; passive; 10	NA; 0; 24.4	Passive; 1-8-1-2-6; severe pain	3 (50.0)
Evangelista et al. [89]	Between-subject; active; 29(17|12)	NA; 0; 27.5	Active; 0-4-4-2-8; severe pain	4 (57.1)
			Active; 0-4-2-2-8; severe pain	
Ferreira-Júnior et al. [90]	Between-subject; active; 45(28|17)	Recreationally trained; 0; 21.2	Active; 0-2-2-2-8; no pain	4 (57.1)
Freitas and Mil-Homens [91]	Between-subject; passive; 10(5|5)	Recreationally trained; NA; 21.2	Passive; 8-1-1-3-8; no pain	1 (14.3)
Junior et al. [92]	Within-subject; active; 9	Sedentary; 0; 25.4	Passive; 0-2-1-2-10; severe pain	4 (66.7)
Konrad and Tilp [93]	Between-subject; passive; 41(20|21)	NA; 29; 23.1	Passive; 0-4-1-5-6; moderate pain	3 (42.9)
Mizuno [94]	Between-subject; passive; 20(11|9)	Recreationally trained; 38; 18.7	Passive; 0-4-1-3-8; no pain	4 (57.1)
Moltubakk et al. [95]	Within-subject; passive; 26	Recreationally trained; 61; 22.0	Active; 1-4-1-7-24; no pain	5 (83.3)
			Active; 1-4-1-7-8; no pain	
			Active; 1-4-1-7-16; no pain	
Nakamura et al. [96]	Between-subject; passive; 18(9|9)	NA; 0; 21.4	Passive; 1-2-1-7-4; severe pain	6 (85.7)
Nakamura et al. [78]	Between-subject; passive; 40(27|13)	Recreationally trained; 0; 20.8	Passive; 0-3-1-4-4; no pain	5 (71.4)
		Recreationally trained; 0; 21.6	Passive; 0-3-1-4-4; moderate pain	
Nakamura et al. [97]	Within-subject; active; 16	Recreationally trained; 0; 21.3	Passive; 0-2-1-2-5; severe pain	5 (83.3)
Nakamura et al. [77]	Within-subject; passive; 28	Sedentary; 0; 20.9	Passive; 1-3-1-3-4; no pain	5 (83.3)
		Sedentary; 0; 21.4	Passive; 1-3-1-3-4; moderate pain	
Panidi et al. [12]	Within-subject; passive; 21	Trained; 100; 13.5	Passive; 1-2-6-5-12; severe pain	5 (83.3)
Peixinho et al. [98]	Between-subject; passive; 20(12|8)	Recreationally trained; 0; 18.9	Mixed; 0-2-2-4-10; moderate pain	5 (71.4)
Simpson et al. [13]	Between-subject; passive; 21(11|10)	NA; 0; 22.0	Passive; 3-1-1-5-3; NA	4 (57.1)
			passive; 3-1-1-5-6; NA	
Warneke et al. [69]	Within-subject; passive; 27	Trained; 41; 27.4	Active; 60-1-1-1-6; moderate pain	4 (66.7)
Warneke et al. [71]	Within-subject; passive; 28	Recreationally trained; 100; 26.9	Active; 60-1-1-1-6; moderate pain	3 (50.0)
		Trained; 0; 27.3	Active; 60-1-1-1-6; moderate pain	
Warneke et al. [99]	Between-subject; passive; 23(23|0)	Recreationally trained; 38; 27.7	Passive; 10-6-1-7-6; severe pain	6 (85.7)
	Within-subject; passive; 23	Recreationally trained; 35; 28.2	Passive; 10-6-1-7-6; severe pain	5 (83.3)
Warneke et al. [100]	Between-subject; passive; 46(23|23)	Recreationally trained; 45; 27.6	Passive; 60-1-1-7-6; moderate pain	6 (85.7)
Wohlann et al. [101]	Within-subject; passive; 22	Recreationally trained; 59; 24.2	Passive; 5-1-4-7-6; severe pain	4 (66.7)
	Within-subject; passive; 44	Recreationally trained; 48; 24.5	Passive; 5-1-4-7-6; severe pain	4 (66.7)
Yahata et al. [102]	Within-subject; passive; 16	Recreationally trained; 0; 21.4	Passive; 1-6-1-2-5; severe pain	4 (66.7)
e Lima et al. [35]	Between-subject; passive; 23(12|11)	Recreationally trained; 0; 19.1	Active; 0-3-1-3-8; severe pain	4 (57.1)

^a^study details presented as comparator (within-subject/between-subject), type of control condition (active/passive), and N (intervention|control)

^b^participant details presented as participant training status, percentage of females in the sample (%), and mean age (years)

^c^intervention details presented as type of static stretching (active/passive/mixed), mean duration of single stretching exercise (s)—number of sets per session (n)—number of different stretching exercises per session (n)—weekly session frequency (n)—intervention period (weeks), stretching intensity (no/moderate/severe pain)

NA = not available

**Table 2:** Detailed information of the static stretching interventions from the included studies

Study	Stretched muscle group	Muscle assessed; assessment method; measure [unit]
Akagi and Takahashi [85]	Plantar flexors	Calf; b-mode ultrasound; muscle thickness [cm]
		Plantar flexor; NA; ROM [°]
Andrade et al. [86]	Plantar flexors	Dorsiflexion; NA; ROM [°]
		Gastrocnemius lateralis; b-mode ultrasound; fascicle length [cm]
		Gastrocnemius lateralis; b-mode ultrasound; muscle thickness [cm]
		Gastrocnemius medialis; b-mode ultrasound; fascicle length [cm]
		Gastrocnemius medialis; b-mode ultrasound; muscle thickness [cm]
Blazevich et al. [87]	Plantar flexors	Gastrocnemius medialis; b-mode ultrasound; fascicle length [mm]
		plantar flexion; NA; ROM [°]
Brusco et al. [88]		NA; NA; ROM [°]
		Biceps femoris; b-mode ultrasound; muscle thickness [mm]
		Semitendinosis; b-mode ultrasound; muscle thickness [mm]
Evangelista et al. [89]	Knee flexors	Biceps femoris; b-mode ultrasound; muscle thickness [mm]
		Rectus femoris; b-mode ultrasound; muscle thickness [mm]
		Triceps surae; b-mode ultrasound; muscle thickness [mm]
		Vastus lateralis; b-mode ultrasound; muscle thickness [mm]
Ferreira-Júnior et al. [90]	Knee flexors	Biceps femoris; b-mode ultrasound; muscle thickness [mm]
Freitas and Mil-Homens [91]	Knee flexors	Biceps femoris; b-mode ultrasound; fascicle length biceps femoris [mm]
		Biceps femoris; b-mode ultrasound; muscle thickness biceps femoris [mm]
Junior et al. [92]	Hip extensors	Hip extension; NA; ROM [°]
		Vastus lateralis; b-mode ultrasound; CSA [cm^2^]
Konrad and Tilp [93]	Plantar flexors	Dorsiflexion; NA; ROM [°]
		Gastrocnemius medialis; b-mode ultrasound; fascicle length [cm]
Mizuno [94]	Plantar flexors	Dorsiflexion; NA; ROM [°]
		Gastrocnemius medialis; b-mode ultrasound; muscle thickness [mm]
Moltubakk et al. [95]	Plantar flexors	Ankle; NA; ROM [°]
		Gastrocnemius medialis; b-mode ultrasound; fascicle length [mm]
		Gastrocnemius medialis; b-mode ultrasound; muscle thickness [mm]
Nakamura et al. [96]	Plantar flexors	Dorsiflexion; NA; dorsiflexion range of motion [°]
		gastrocnemius medialis; b-mode ultrasound; fascicle length [cm]
Nakamura et al. [78]	Plantar flexors	Dorsiflexion; NA; ROM [°]
		Gastrocnemius lateralis; b-mode ultrasound; fascicle length [mm]
		Gastrocnemius lateralis; b-mode ultrasound; muscle thickness [mm]
		Gastrocnemius medialis; b-mode ultrasound; fascicle length [mm]
		Gastrocnemius medialis; b-mode ultrasound; muscle thickness [mm]
		Soleus; b-mode ultrasound; muscle thickness [mm]
Nakamura et al. [97]	Hip extensors	Gluteus maximus; b-mode ultrasound; muscle thickness [mm]
		Hamstring; b-mode ultrasound; muscle thickness [mm]
		Rectus femoris; b-mode ultrasound; muscle thickness [mm]
		Vastus intermedialis; b-mode ultrasound; muscle thickness [mm]
		Vastus lateralis; b-mode ultrasound; muscle thickness [mm]
		Vastus medialis; b-mode ultrasound; muscle thickness [mm]
Nakamura et al. [77]	Plantar flexors	Dorsiflexion; NA; ROM [°]
		Gastrocnemius lateralis; b-mode ultrasound; fascicle length [mm]
		Gastrocnemius lateralis; b-mode ultrasound; muscle thickness [mm]
		Gastrocnemius medialis; b-mode ultrasound; fascicle length [mm]
		Gastrocnemius medialis; b-mode ultrasound; muscle thickness [mm]
		Soleus; b-mode ultrasound; muscle thickness [mm]
Panidi et al. [12]	Plantar flexors	Dorsiflexion; NA; ROM [°]
		Gastrocnemius lateralis; b-mode ultrasound; CSA [cm^2^]
		Gastrocnemius lateralis; b-mode ultrasound; fascicle length [cm]
		Gastrocnemius medialis; b-mode ultrasound; CSA [cm^2^]
		Gastrocnemius medialis; b-mode ultrasound; fascicle length [cm]
		Gastrocnemius; b-mode ultrasound; CSA [cm^2^]
Peixinho et al. [98]	Plantar flexors	Gastrocnemius medialis; b-mode ultrasound; CSA [mm^2^]
		Gastrocnemius medialis; b-mode ultrasound; fascicle length [cm]
		Max dorsiflexion ankle; NA; ROM [°]
Simpson et al. [13]	Plantar flexors	Gastrocnemius lateralis; b-mode ultrasound; fascicle length [cm]
		Gastrocnemius lateralis; b-mode ultrasound; muscle thickness [cm]
		Gastrocnemius medialis; b-mode ultrasound; fascicle length [cm]
		Gastrocnemius medialis; b-mode ultrasound; muscle thickness [cm]
		Gastrocnemius; b-mode ultrasound; muscle thickness [cm]
Warneke et al. [69]		NA; NA; knee to wall [cm]
		Gastrocnemius lateralis; b-mode ultrasound; muscle thickness [mm]
Warneke et al. [71]		NA; NA; ROM [°]
		NA; NA; knee to wall [cm]
		Gastrocnemius lateralis; b-mode ultrasound; muscle thickness [mm]
		Gastrocnemius medialis; b-mode ultrasound; muscle thickness [mm]
Warneke et al. [99]	Plantar flexors	Dorsiflexion; NA; ROM [°]
		Dorsiflexion; NA; knee to wall [°]
		Gastrocnemius lateralis; b-mode ultrasound; CSA [mm^2^]
		Gastrocnemius lateralis; b-mode ultrasound; muscle thickness [mm]
		Gastrocnemius medialis; b-mode ultrasound; CSA [mm^2^]
		Gastrocnemius medialis; b-mode ultrasound; muscle thickness [mm]
Warneke et al. [100]	Plantar flexors	Dorsiflexion; NA; ROM [°]
		Dorsiflexion; NA; knee to wall [°]
		Gastrocnemius lateralis; b-mode ultrasound; muscle thickness [mm]
		Gastrocnemius medialis; b-mode ultrasound; muscle thickness [mm]
Wohlann et al. [101]	Hip extensors	Hip flexion; NA; ROM [°]
		Knee flexor; NA; Thomas test (knee) [°]
		Rectus femoris; b-mode ultrasound; muscle thickness [mm]
Yahata et al. [102]	Plantar flexors	Gastrocnemius lateralis; b-mode ultrasound; fascicle length [mm]
		Gastrocnemius lateralis; b-mode ultrasound; muscle thickness [mm]
		Gastrocnemius medialis; b-mode ultrasound; fascicle length [mm]
		Gastrocnemius medialis; b-mode ultrasound; muscle thickness [mm]
		Soleus; b-mode ultrasound; muscle thickness [mm]
e Lima et al. [35]	Knee flexors	Biceps femoris; b-mode ultrasound; muscle thickness [mm]
		knee extension; NA; ROM [°]
		knee flexion; NA; ROM [°]
		vastus lateralis; b-mode ultrasound; muscle thickness [mm]

CSA, cross sectional area; ROM, range of motion; NA, Not available

### Main Models—All Effects

The outcomes remained consistent for both muscle hypertrophy (e.g., CSA) and muscle architecture (e.g., fascicle length). Therefore, we performed the analysis considering all measures of muscle hypertrophy and architecture together. The main model (118 effect sizes across 25 clusters [median = 4, range 1 to 14 outcomes per cluster]) revealed an unclear effect with a trivial point estimate (SMD = 0.118 [95% CI = 0.023 to 0.213; 95% PI = − 0.233 to 0.469; *p* = 0.017]) and low heterogeneity (I^2^ = 24% [I^2^-between = 24%, I^2^-within = 0%]).

**Fig. 2:**
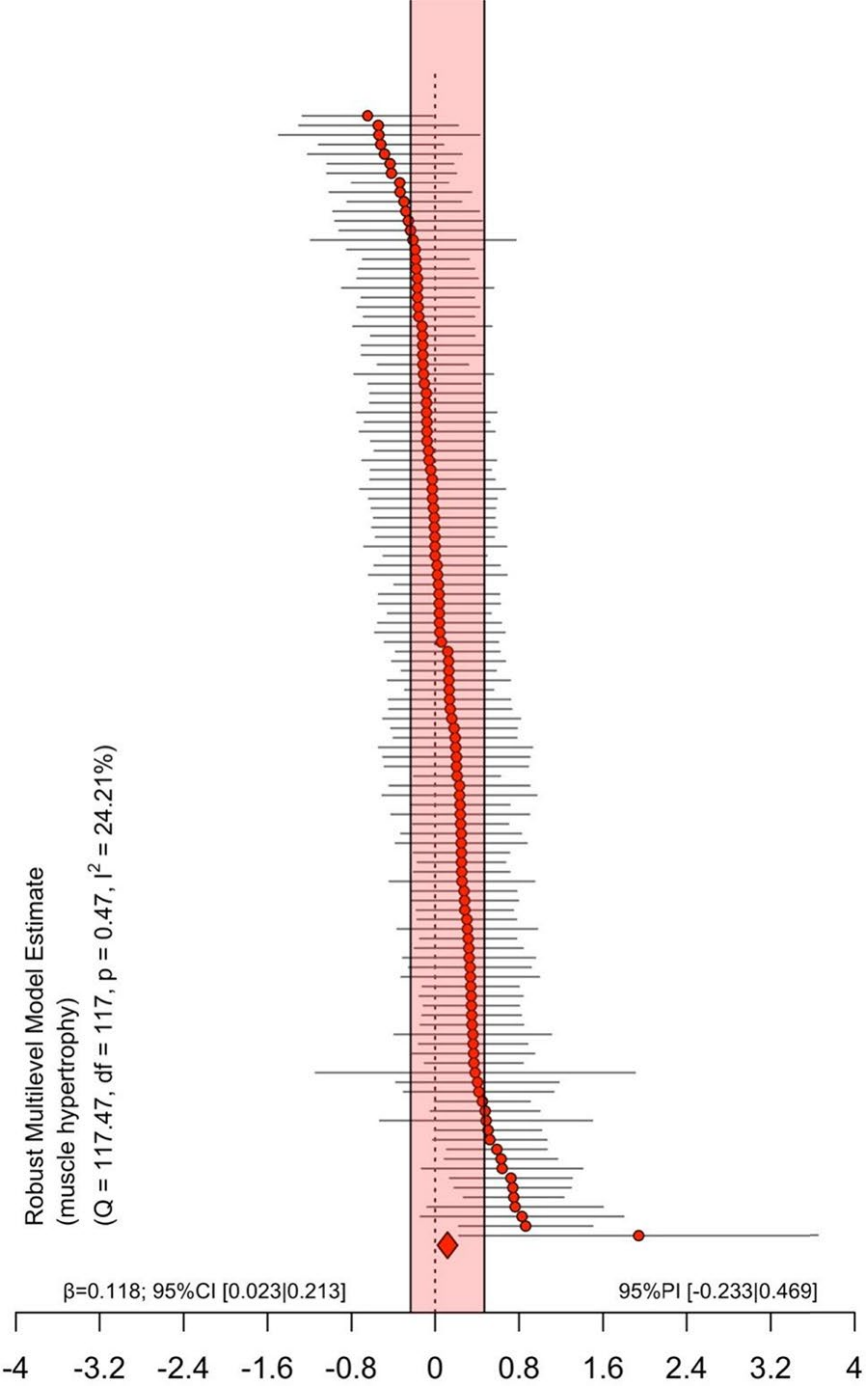
Ordered caterpillar plot of all effects for muscle hypertrophy including prediction intervals. 95% PI = 95% Prediction interval, 95% CI = 95% Confidence interval

**Fig. 3:**
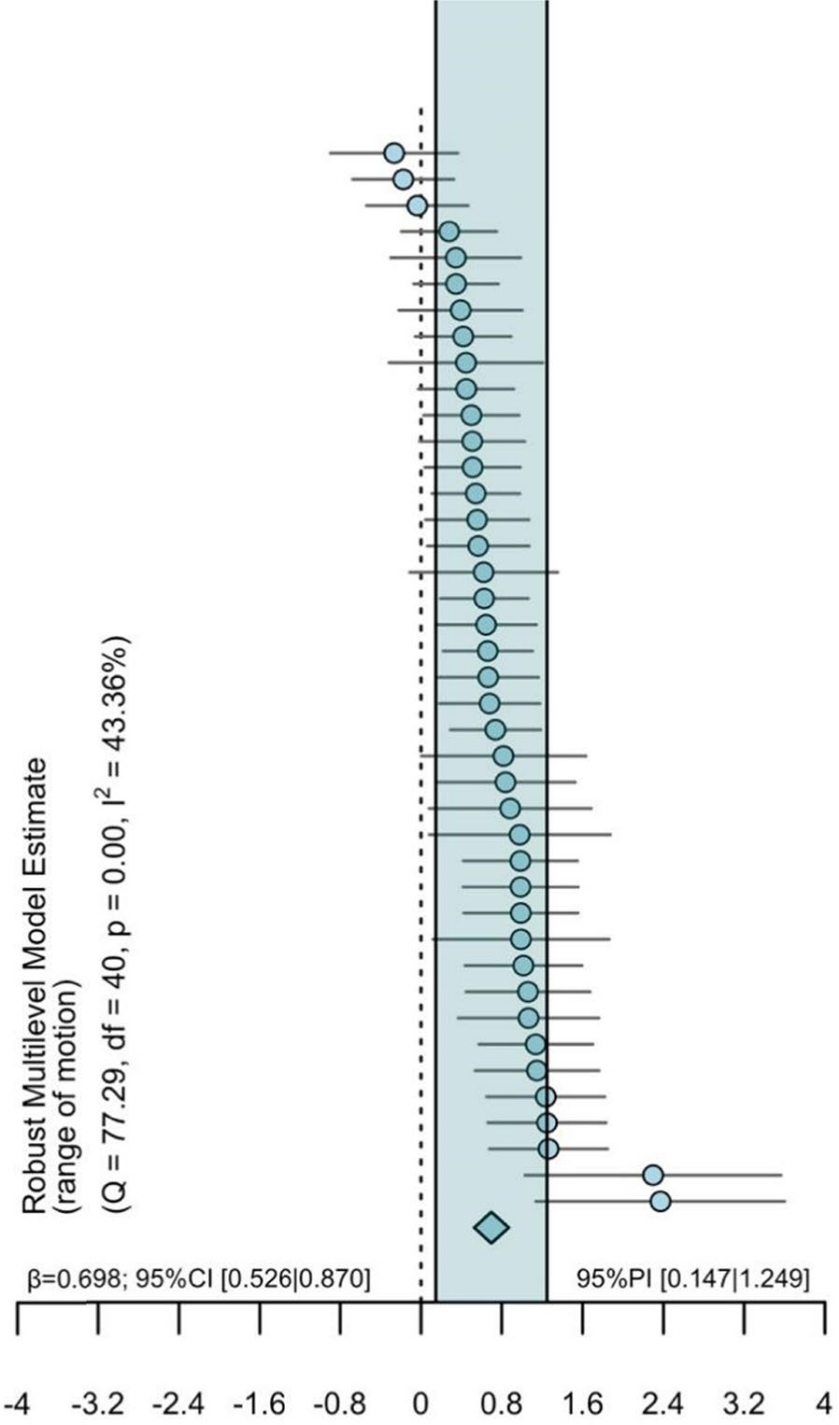
Ordered caterpillar plot of all effects for joint range of motion including prediction intervals. 95% PI = 95% Prediction interval, 95% CI = 95% Confidence interval

The model for joint ROM (41 effect sizes across 19 clusters [median = 1, range 1 to 6 outcomes per cluster]) revealed a clear positive effect with a moderate point estimate (SMD = 0.698 [95% CI = 0.526 to 0.870; 95% PI = 0.147 to 1.249; *p* < 0.001]) and moderate heterogeneity (I^2^ = 43% [I^2^-between = 43%, I^2^-within = 0%]) (Figs. 2 and 3).

**Fig. 4:**
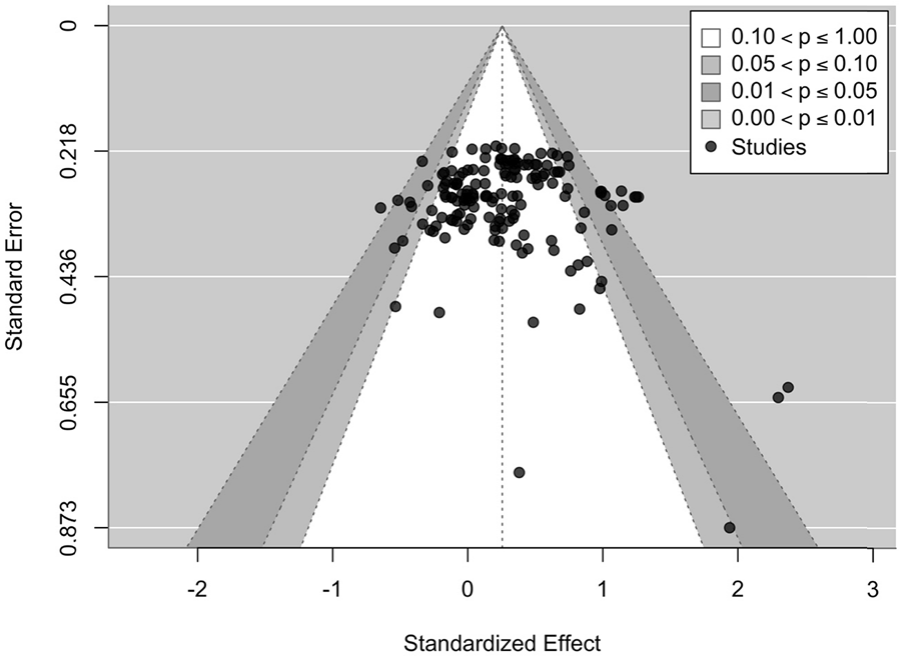
Contour enhanced funnel plot

**Fig. 5:**
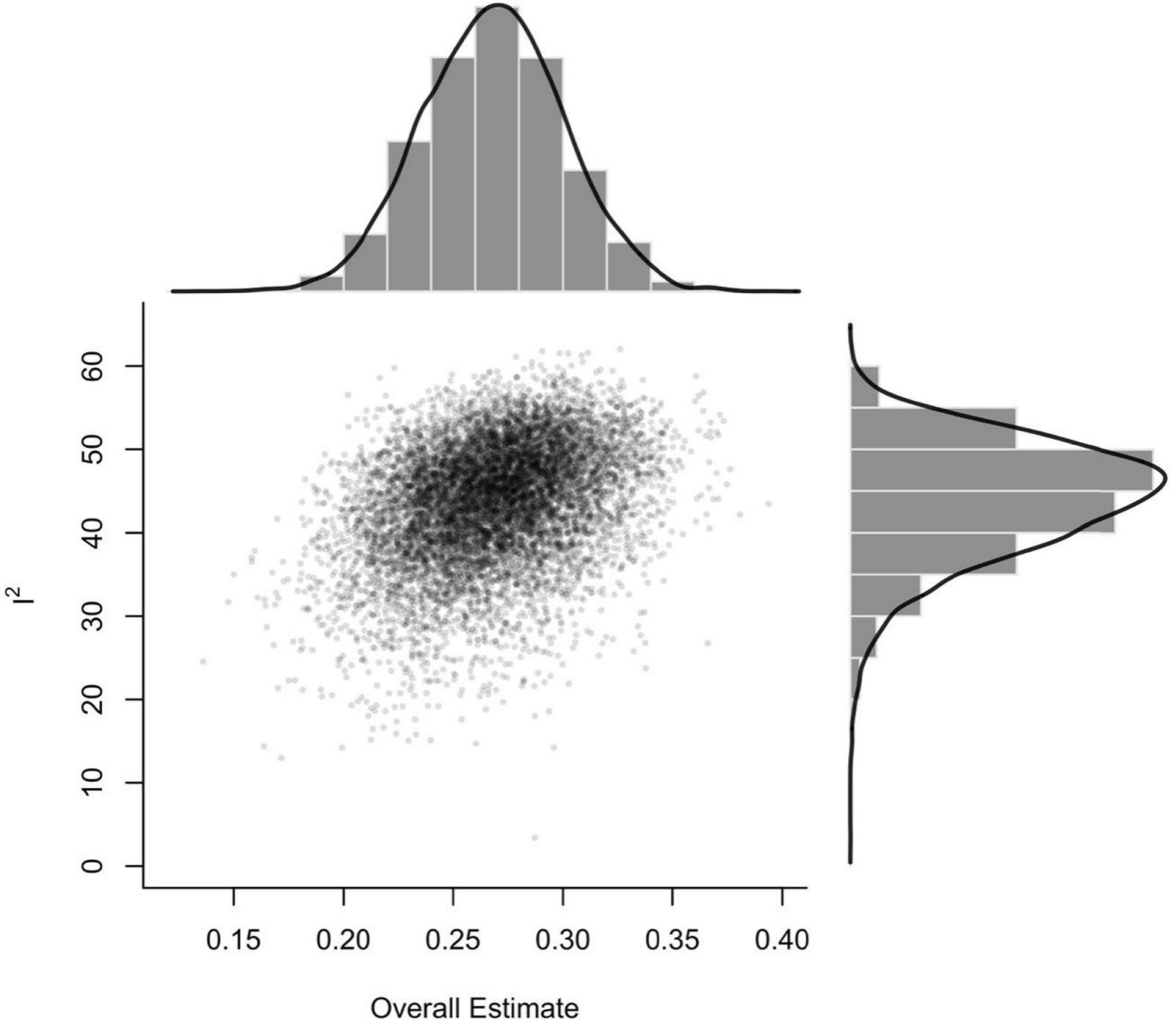
Graphical display of study heterogeneity (GOSH) plot (10,000 Samples)

**Fig. 6:**
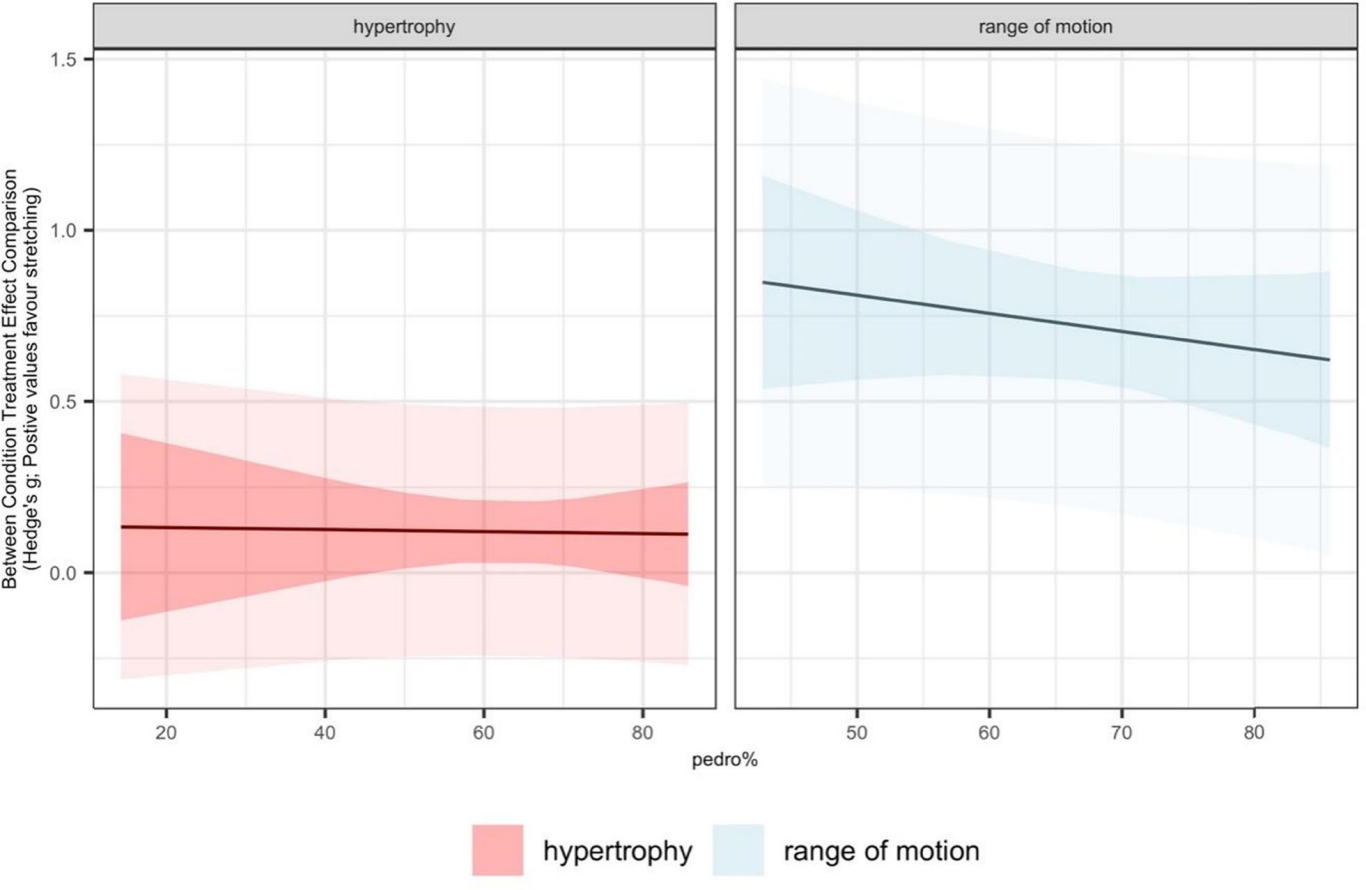
Meta-analytic regression plot of PEDro scores (%) for muscle hypertrophy including prediction intervals. pedro% = ratio of achieved PEDro scores to all applicable PEDro scores

Visual inspection of funnel and GOSH plots indicated a seemingly symmetrical distribution pattern of the effects that might reflect an absence of publication bias (Figs. 4 and 5). Further, meta-regression analysis showed no clear evidence that outcomes were predicted by the PEDro percent for hypertrophy and joint ROM (hypertrophy: β = − 0.000 [95% CI = − 0.006 to 0.005]; p = 0.911; joint ROM: β = − 0.005 [95% CI = − 0.016 to 0.006]; *p* = 0.323) (Fig. 6).

### Subgroup and Meta-Regression Analyses

Likelihood ratio tests (LRT) for subgroup analysis related to hypertrophy indicated that incorporating participants’ training status into the model improved the model’s fit (Table 3). Specifically, results indicated that chronic SS exercises induced an unclear effect on muscle hypertrophy with a trivial negative point estimate for sedentary participants (β = − 0.081; [95% CI = − 0.222 to 0.059; 95% PI = − 0.399 to 0.236], *p* = 0.236), an unclear effect with a trivial positive point estimate for recreationally trained participants (β = 0.115; [95% CI = − 0.007 to 0.236; 95% PI = − 0.195 to 0.425]; *p* = 0.062), and a clear effect with small positive point estimate for trained participants (β = 0.424; [95% CI = 0.260 to 0.588; 95% PI = 0.095 to 0.753]; *p* = 0.001).

**Table 3:** Subgroup analysis using the log-likelihood-ratio tests

Subgroup	Outcome	AIC	BIC	*p*-value
Participants training status				
	Hypertrophy	17.16	29.60	0.013
		21.85	29.32	
	Range of motion	40.10	48.16	0.493
		37.52	42.35	
Stretching intensity				
	Hypertrophy	30.75	43.97	0.970
		26.81	34.74	
	Range of motion	48.63	57.19	0.293
		47.08	52.22	
Study design				
	Hypertrophy	42.47	53.55	0.817
		40.52	48.84	
	Range of motion	47.91	54.77	0.280
		47.08	52.22	
Type of control group				
	Hypertrophy	42.52	53.61	0.998
		40.52	48.84	
Type of static stretching				
	Hypertrophy	40.31	51.29	0.472
		38.83	47.06	
	Range of motion	48.10	54.85	0.388
		46.84	51.91	
Assessed muscle group				
	Hypertrophy	44.89	58.70	0.990
		40.91	49.19	
	Range of motion	47.82	55.46	0.251
		46.59	51.17	
Muscle architecture				
	Hypertrophy	40.81	51.90	0.191
		40.52	48.84	

AIC, Akaike Information Criterion; BIC, Bayesian Information Criterion

**Fig. 7:**
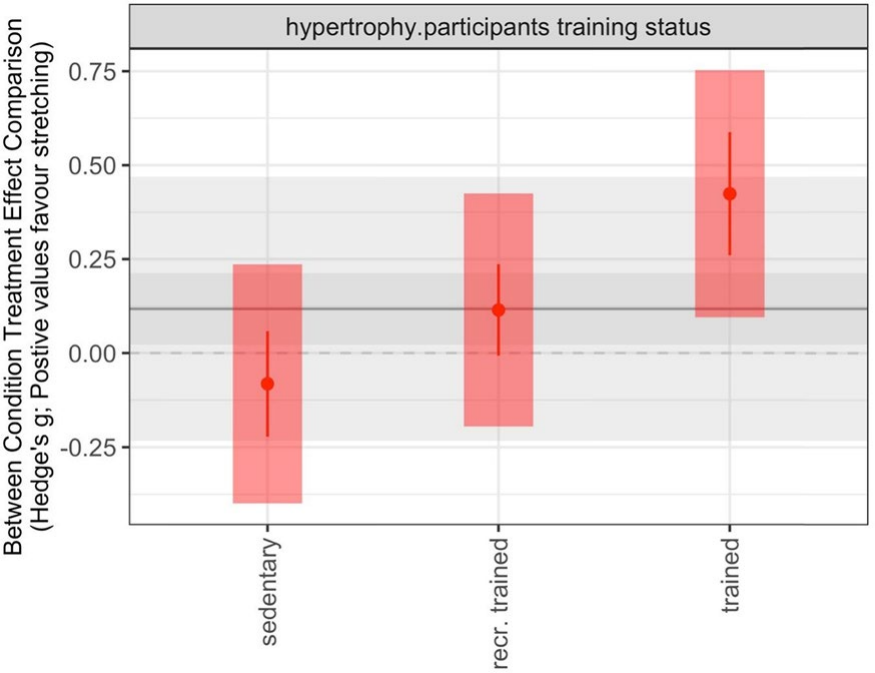
Subgroup-plots of categorical subgroups on muscle hypertrophy including prediction intervals. Main models point estimate, confidence intervals, and prediction intervals are plotted in the background using grey lines/shading to allow comparison. recr. = recreationally

All subgroups analyses are presented in Table 4 and Fig. 7.

**Table 4:** Results of the subgroup analyses including effect sizes, confidence and prediction intervals of the factors displaying a moderating effect on muscle hypertrophy

Subgroup	Training level	Beta	CI	PI	*p*-value	*p*-value between	I^2*
Participants ‘ training status							
	Sedentary	− 0.081	[− 0.222|0.059]	[− 0.399|0.236]	0.236	0.001	19 (19,0)
	Recreationally trained	0.115	[− 0.007|0.236]	[− 0.195|0.425]	0.062		
	Trained	0.424	[0.260|0.588]	[0.095|0.753]	0.000		

CI, confidence interval; PI, prediction interval *reported as I^2 overall (I^2 between, I^2 within)

Further, the meta-regression analyses revealed that the chronic effect of SS on muscle hypertrophy is moderated by the number of different SS exercises (β = 0.069, [95% CI = 0.041 to 0.097]; *p* < 0.001) with more variety of stretching exercises eliciting larger increases in muscle hypertrophy, the mean duration of single stretching exercise (β = 0.006, [95% CI = 0.002 to 0.010]; *p* = 0.008)^1^ with longer mean duration of single stretching exercise inducing larger hypertrophic gains, as well as the time under stretching per session (β = 0.006, [95% CI = 0.003 to 0.009]; *p* = 0.001), per week (β = 0.001, [95% CI = 0.000 to 0.001]; *p* = 0.001) and in total (β = 0.008, [95% CI = 0.003 to 0.013]; *p* = 0.001) with longer times inducing larger gains in muscle hypertrophy. Interestingly, the LRT revealed an improvement of the model fit for the percentage of females in sample (*p* = 0.032), however, the model showed no clear effect but indicated a positive trend for the percentage of females in a sample (β = 0.003, [95% CI = − 0.000 to 0.005]; *p* = 0.058) with higher percentages of females inducing larger gains in hypertrophy. No clear effects on hypertrophy could be found for the remaining variables (i.e., mean age, weekly session frequency, intervention period) (Table 5).

**Table 5:** Results of the meta-regression analysis using the log-likelihood ratio tests

Subgroups	Outcomes	AIC	BIC	*p*-value
Proportion of females				
	Hypertrophy	35.83	46.84	0.032
		38.44	46.70	
	Range of motion	48.86	55.71	0.636
		47.08	52.22	
Mean age				
	Hypertrophy	42.03	53.12	0.483
		40.52	48.84	
	Range of motion	48.45	55.30	0.426
		47.08	52.22	
Intervention period				
	Hypertrophy	42.48	53.56	0.836
		40.52	48.84	
	Range of motion	48.82	55.68	0.612
		47.08	52.22	
Weekly session frequency				
	Hypertrophy	42.46	53.54	0.802
		40.52	48.84	
	Range of motion	48.88	55.73	0.653
		47.08	52.22	
Mean duration of single stretching exercise				
	Hypertrophy	35.87	46.95	0.010
		40.52	48.84	
	Range of motion	47.49	54.34	0.207
		47.08	52.22	
Number of repetitions per exercise				
	Hypertrophy	40.10	51.19	0.120
		40.52	48.84	
	Range of motion	48.55	55.40	0.464
		47.08	52.22	
Number of repetitions per session				
	Hypertrophy	41.67	52.75	0.355
		40.52	48.84	
	Range of motion	49.02	55.87	0.804
		47.08	52.22	
Total repetitions number				
	Hypertrophy	41.79	52.87	0.391
		40.52	48.84	
	Range of motion	48.92	55.77	0.688
		47.08	52.22	
Number of different stretching exercises				
	hypertrophy	36.78	47.86	0.017
		40.52	48.84	
	range of motion	42.37	49.22	0.010
		47.08	52.22	
Stretching duration per session				
	Hypertrophy	32.70	43.78	0.002
		40.52	48.84	
	Range of motion	47.74	54.59	0.246
		47.08	52.22	
Weekly stretching duration				
	Hypertrophy	37.30	48.38	0.022
		40.52	48.84	
	Range of motion	48.37	55.23	0.400
		47.08	52.22	
Total stretching duration				
	Hypertrophy	36.90	47.99	0.018
		40.52	48.84	
	Range of motion	48.51	55.36	0.448
		47.08	52.22	

AIC, Akaike Information Criterion; BIC, Bayesian Information Criterion

For joint ROM, LRT revealed a model fit improvement for the number of different stretching exercises (β = 0.175, [95% CI = 0.067 to 0.284]; *p* = 0.003) with a greater variety of stretching exercises eliciting larger increases (Table 5). No clear effects on joint ROM could be found for the remaining variables (i.e., percentage of females in sample, mean age, weekly session frequency, intervention period, SS duration per session, per week, and in total, mean duration of single SS exercise, number of repetitions per exercise).

**Fig. 8:**
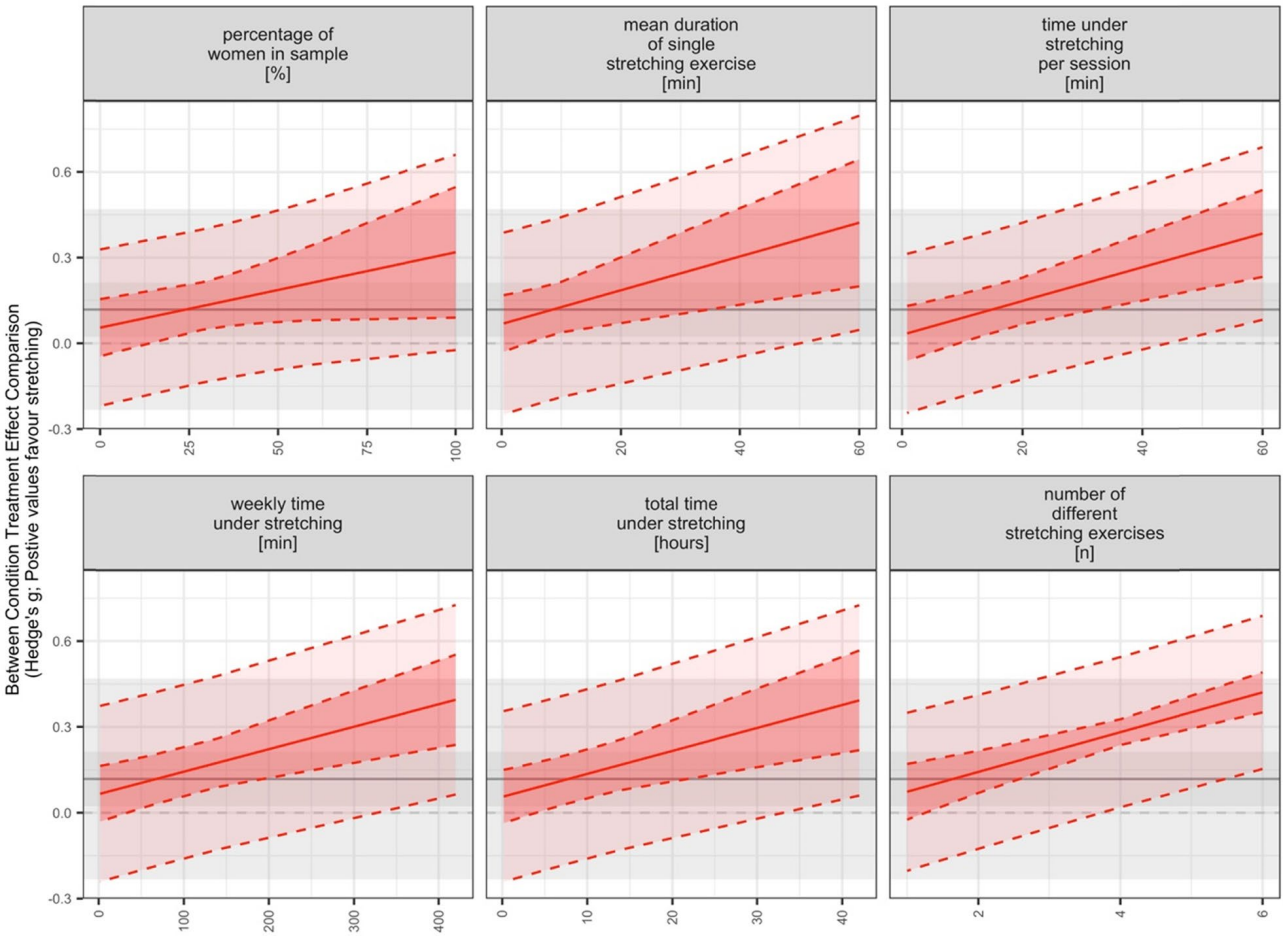
Meta-analytic plots of continuous subgroups on muscle hypertrophy including prediction intervals. Main models point estimate, confidence intervals, and prediction intervals are plotted in the background using grey lines/shading to allow comparison

**Fig. 9:**
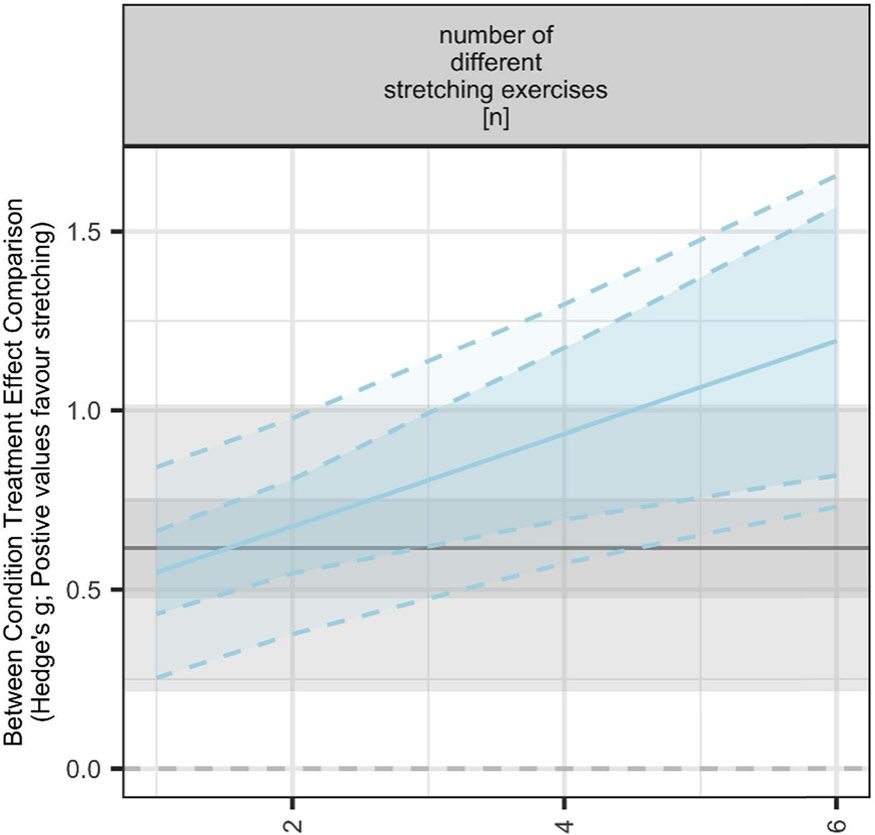
Meta-analytic plots of continuous subgroups on joint range of motion including prediction intervals. Main models point estimate, confidence intervals, and prediction intervals are plotted in the background using grey lines/shading to allow comparison

**Fig. 10:**
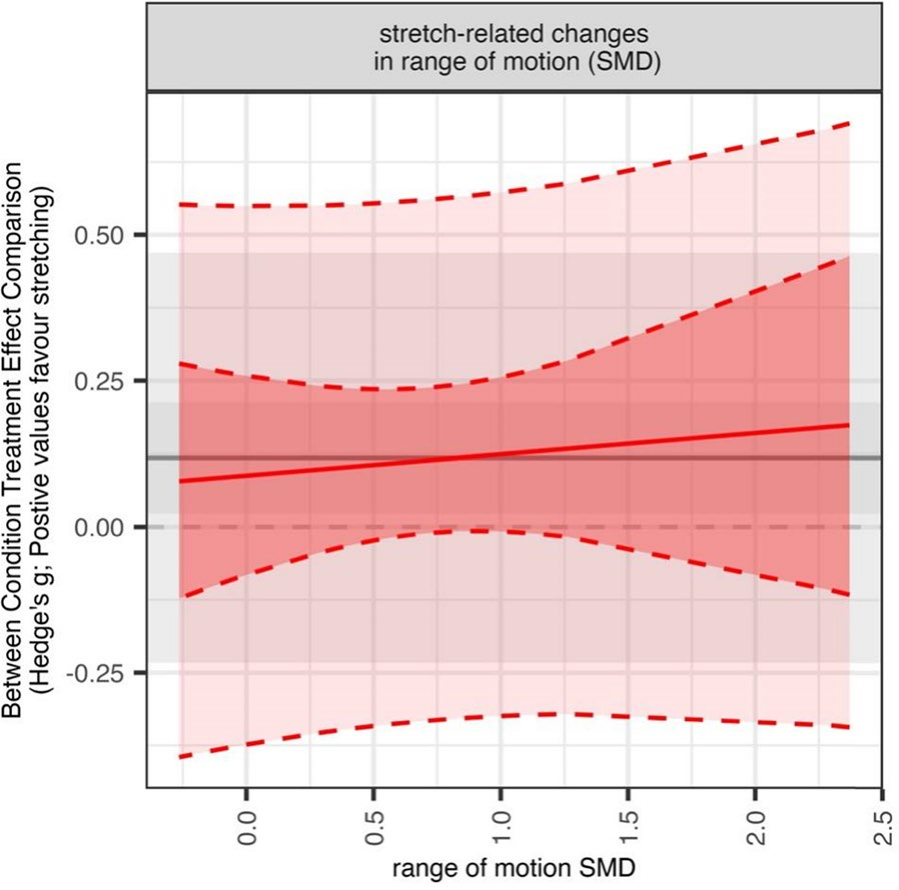
Meta-analytic plots of stretch-related gains in joint range of motion on hypertrophy including prediction intervals. Main models point estimate, confidence intervals, and prediction intervals are plotted in the background using grey lines/shading to allow comparison. SMD, Standardised Mean Difference

No statistically significant associations between SS-related joint ROM improvements and increases in muscle hypertrophy were revealed (β = 0.036, [95% CI = − 0.123 to 0.196]; *p* = 0.638). Further details related to the meta-regression analyses are displayed in Table 6 and Figs. 8, 9 and 10.

**Table 6:** Results of the meta-regression analysis including the effect sizes and confidence intervals of the factors displaying a moderating effect

Measures	Subgroups	Beta	CI	*p*-value	I^2*
Range of motion					
	Number of different stretching exercises	0.175	[0.067|0.284]	0.003	55 (55,0)
Hypertrophy					
	% females in the sample	0.003	[− 0.000|0.005]	0.058	15 (4,11)
	Mean duration of single stretching exercise	0.006	[0.002|0.010]	0.008	23 (23,0)
	Stretching duration per session	0.006	[0.003|0.009]	0.001	16 (16,0)
	Weekly stretching duration	0.001	[0.000|0.001]	0.001	21 (21,0)
	Total stretching duration	0.008	[0.003|0.013]	0.001	19 (19,0)
	Number of different stretching exercises	0.069	[0.041|0.097]	0.000	14 (14,0)
	Stretch-related improvements in range of motion	0.036	[− 0.123|0.196]	0.638	39 (39,0)

CI, confidence interval. *Reported as I^2 overall (I^2 between, I^2 within)

## Discussion

This study aimed to systematically review and meta-analyse the literature related to the chronic effect of SS exercises on skeletal muscle hypertrophy in healthy individuals. The overall findings pointed toward an unclear chronic effect of SS exercises on muscle hypertrophy, although the range of the 95% PI reveals that the chance of a positive effect from SS training exceeds that of a negative effect. Subgroup analysis indicated that trained individuals achieved greater hypertrophy compared to their recreationally trained and sedentary counterparts. The meta-regression analyses indicated marginally greater benefits in samples with a higher proportion of females. Additionally, larger skeletal muscle hypertrophy effects seemed to be induced by a more varied selection of SS exercises, longer single stretch durations, and increased time under SS per session, per week, and in total. Moreover, results indicate that the gain in joint ROM is not associated with changes in muscle hypertrophy.

### The Chronic Effect of Static Stretching on Muscle Hypertrophy

Emerging evidence indicates positive effects of long-term SS training on muscle strength and power [10, 69–71]. For example, the results of a recent systematic review with meta-analysis on the chronic effects of SS exercises on muscle strength and power in healthy individuals showed beneficial effects, though trivial-to-small in magnitude [9]. Similarly, the authors of another recent meta-analysis reported a small positive effect of long-term SS training on muscle strength in healthy individuals [10]. Speculations around the potential underpinning mechanisms of muscle strength and power gains included SS training-inducing muscle hypertrophy, among others [12, 13, 71]. However, the main finding of this study does not seem to support this assumption, as the aggregated data of the 25 included studies indicated an overall unclear effect of SS training on muscle hypertrophy with low observed heterogeneity. Of note, this study distinguishes itself from the recently published meta-analysis [36] by its unique approach as it relies on the PI to interpret the results [37, 38]. In fact, PI is a powerful tool to inform about between-study heterogeneity and, in contrast to I^2^, has the advantage to display heterogeneity in the same metric as the original effect size [37, 38]. Therefore, the omission of reporting PI might imply the recommendation of treatments with an insufficient evidence base or the potential for harm in practical applications [38]. In the current study, the 95% PI does overlap the zero line, indicating that future similar studies would show results ranging from a small negative to a nearly moderate positive effect on muscle hypertrophy, supporting the overall uncertain effect. However, it is worth noting that the range of the 95% PI indicates that the chance for a positive effect following SS training exceeds that of a negative effect. Of note, we conducted separate analyses of the effects of SS on muscle hypertrophy (e.g., CSA) and architecture (e.g., fascicle length) and observed consistent findings. There is evidence that fascicle length serves as an indicator of muscle hypertrophy [72–76]. Hence, we carried out the analysis by considering outcomes of both muscle CSA and fascicle length together.

The current finding is in line with previous studies [23, 77, 78], although other studies reported muscle hypertrophy following SS training [12, 13, 71]. In their narrative review of the literature, Nunes et al. [23] argued that changes in muscle size and architecture do not seem to be stimulated by passive, low-intensity stretching. However, the authors speculated that muscle hypertrophy could occur under particular conditions, such as when stretching with a high level of intensity to produce sufficient tensile strain [23]. The findings of a recent systematic review with meta-analysis [36] indicated trivial-to-small increases in fascicle length at rest and during stretching following chronic SS exercises with no effects on muscle thickness in healthy individuals. However, these results should be interpreted with caution as the authors did not report PIs, which may weaken the assessment of between-study heterogeneity’s influence on the primary conclusions [37]. Consequently, this could lead to misinterpretation of the findings and potentially result in misleading conclusions. To ensure a more robust and comprehensive understanding of the effects of chronic SS exercises on muscle architecture, future studies should prioritize reporting PIs to account for potential heterogeneity and increase the validity of the results. Overall, based on the findings of the current study it seems that SS training leads to an overall unclear effect on muscle hypertrophy in healthy individual, although interpretation across the range of PI suggests a potential modest beneficial effect.

### Subgroup and Meta-Regression Analysis

Subgroup analyses revealed that training status moderated the effects on hypertrophy, with trained individuals (β = 0.424; 95% PI = 0.095 to 0.753) displaying larger effects compared to recreationally trained individuals (β = 0.115; 95% PI = − 0.195 to 0.425) and sedentary individuals (β = − 0.081; 95% PI = − 0.399 to 0.236). This suggests that the chronic effect of SS on skeletal muscle hypertrophy progressively increases with increasing training status. The reported PI provides support for this claim as it indicates that future similar studies would display trivial to moderate positive effects of SS exercises on muscle hypertrophy in trained individuals. Notably, both ends of the PIs for sedentary and recreationally active individuals overlap zero, indicating that future similar studies may yield inconsistent findings ranging from trivial to small negative or positive effects. However, for trained individuals, both ends of the PI are above the zero line, implying that chronic SS exercises consistently lead to positive effects on skeletal muscle hypertrophy in this group. These observations were unexpected, considering the law of diminishing returns. While the exact mechanisms responsible for this phenomenon remain undetermined, one could speculate that because trained individuals are accustomed to intense training regimens, they may have a higher tolerance for the stretching stimulus, resulting in the performance of higher intensity stretch training. This could have resulted in more substantial hypertrophy [23]. Future research is needed to elucidate the exact mechanisms underpinning the greater hypertrophic response following SS training in those with more training experience.

Additionally, the meta-regression indicated the potential for larger gains in skeletal muscle hypertrophy in samples that included higher percentages of females (β = 0.003, [95% CI = − 0.000 to 0.006]; p = 0.058). The meta-analysis by Arntz et al. [9] also revealed that higher proportions of females amplified the chronic effects of SS on muscle strength. The seemingly greater effect of SS training on muscle hypertrophy in samples with higher percentages of females is not consistent with the existing, albeit limited literature [71]. In fact, it is well-known that females have better joint ROM than males [79]. As a result, it seems to be more challenging for females to achieve sufficient stretch intensity [71]. This, in turn, may lead to a diminished mechanical stimulus on the stretched muscle compared to males, potentially impacting hypertrophic adaptation [71]. This finding was supported by the study of Warneke et al. [71] who revealed greater increase in muscle thickness of the gastrocnemius in males compared to females following six weeks of training consisting of one-hour SS exercises per day. The difference between the present and existing knowledge [71] could not be fully explained. Although speculative, differences in trainability between the sexes could potentially have influenced the outcomes. In general, females tend to be less active than males. This can be attributed to the historically systematic exclusion of females from organized sports [80, 81] and restricted access to sports and physical activities [82]. It is conceivable that even a relatively low mechanical stimulus level could trigger hypertrophic adaptation in less active females. Overall, the current outcomes should be interpreted with caution. Because sex-specific differences in hypertrophic responses to SS exercises have not been sufficiently investigated in the existing literature [71], additional research is needed to provide more insights into this topic.

Further, results indicated that the effects of chronic SS exercises on muscle hypertrophy is moderated by the number of different SS exercises with greater variety of SS exercises generating larger gains in muscle hypertrophy (β = 0.069, [95% CI = 0.041 to 0.097]; *p* < 0.001). The mean duration of a single stretching exercise constitutes another moderator variable with longer durations inducing larger muscle hypertrophy improvements (β = 0.006, [95% CI = 0.002 to 0.010]; *p* = 0.008). Moreover, the time under stretching per session (β = 0.006, [95% CI = 0.003 to 0.009]; *p* = 0.001), per week (β = 0.001, [95% CI = 0.000 to 0.001]; *p* = 0.001) and in total (β = 0.008, [95% CI = 0.003 to 0.013]; *p* = 0.001) represent additional mediating factors with longer times leading to larger gains in muscle hypertrophy. Arntz et al. [9] reported that the chronic effects of SS on muscle strength were moderated by the number of repetitions per stretching exercise and session (β = 0.023, *p* = 0.004 and β = 0.013, *p* = 0.008, respectively), with more repetitions associated with larger muscle strength improvements. Overall, this leads us to conclude that increasing SS duration appears to be decisive in stimulating hypertrophic gains. Practically speaking, it seems that SS exercises may not be highly effective in enhancing skeletal muscle hypertrophy unless a higher duration of training is employed. These findings could contribute to the reshaping of effective training prescriptions. These results confirm those reported by Warneke et al. [11], who concluded that high SS volumes should be used to stimulate skeletal muscle hypertrophy. However, to improve our understanding, future studies should focus on the underpinning mechanisms and optimal dose–response analysis.

### The Chronic Effect of Static Stretching on Joint Range of Motion

Regarding joint ROM, findings indicate a clear positive effect with a moderate point estimate (SMD = 0.698; 95% PI = 0.147 to 1.249; *p* < 0.001) and moderate heterogeneity (I^2^ = 43%). The PI suggests positive effects ranging from small to large. These findings provide robust evidence for the beneficial impact of chronic SS on joint ROM across healthy populations. Similar to our muscle hypertrophy subgroup analysis findings, meta-regression analysis revealed a moderating effect of the number of different stretching exercises (β = 0.175, [95% CI = 0.067 to 0.284]; *p* = 0.003), with a greater variety of stretching exercises eliciting larger increases in joint ROM. This suggests that the duration of SS is a crucial factor, influencing not only hypertrophy but also gains in joint ROM. In a previous meta-analysis, Arntz et al. [9] revealed larger increases in joint ROM with more repetitions per session (β = 0.094, *p* = 0.016), more time under stretching per session (β = 0.090, *p* = 0.026), and more total time under stretching (β = 0.078, *p* = 0.034).

Chronic SS exercises are believed to lead to joint ROM gains primarily via two underlying mechanisms. The most widely accepted theory is based on the sensory perception theory, which suggests that prolonged exposure to stretching enhances stretch tolerance [83]. The second mechanism is known as the mechanical theory, which relates to potential changes in the mechanical properties (i.e., decreased tissue stiffness) of the muscle–tendon unit or alterations in its geometry (e.g., increased number of sarcomeres in series and increased length of the fascicle) following chronic stretching exercises [83, 84]. It remains to be determined the extent to which these mechanisms influence results across populations.

Furthermore, the findings indicate that the gain in joint ROM does not coordinate changes in muscle hypertrophy. More specifically, no statistically significant associations between SS-related joint ROM improvements and increases in muscle hypertrophy were revealed (β = 0.036; 95% CI = − 0.123 to 0.196; *p* = 0.638). This highlights that any increase in muscle hypertrophy appears to be independent of gains in joint ROM. However, this finding is preliminary, and further research is warranted to delve deeper into this aspect and gather more insightful evidence.

### Limitations

Some limitations of this systematic review with meta-analysis should be acknowledged. First, subgroups and meta-regression analyses were computed independently, not accounting for potential interactions between factors. Second, meta-regression analysis of the effect of study quality on joint ROM outcomes revealed a significant negative effect. This means that larger effect sizes are more likely to be found in lower quality studies, leading to a potential overestimation of the magnitude of the effects of SS on joint ROM in this study. Therefore, the results presented herein pertaining to joint ROM must be interpreted with caution.

## Conclusions

The outcomes of this study highlight a prevailing ambiguity concerning the chronic effects of SS exercises on skeletal muscle hypertrophy. However, given that the lower bound PI shows the potential for a small negative effect while the upper bound PI shows the potential for an almost moderate positive effect, the findings do suggest a potential hypertrophic benefit of SS, which may be dependent on certain factors. Specifically, subgroup analysis suggests that trained individuals demonstrate more substantial enhancements when contrasted with their recreationally trained and sedentary counterparts. The meta-regression analyses also point to potential greater hypertrophic effects within groups characterized by a higher proportion of females. However, the practical significance of this finding is dubious. Moreover, several variables seemed to enhance SS-induced skeletal muscle hypertrophy including a greater variety of SS exercises, longer mean duration of single stretching exercise, and an increased cumulative time under SS per session, per week, and overall. Furthermore, the findings indicate that the gain in joint ROM does not correlate with the change in muscle hypertrophy. From a practical standpoint, it appears that the efficacy of SS exercises in promoting long-term skeletal muscle hypertrophy may necessitate a higher training duration.

## Data Availability

The datasets generated during and/or analysed during the current study as well as supplementary materials are available in the Open Science Framework (OSF) repository. All documents can be consulted at the following link: https://osf.io/snzba/.

## Abbreviations

SS: Static stretching
ROM: Range of motion
CSA: Cross-sectional area
PI: 95% prediction interval
CI: 95% confidence interval
PRISMA: Preferred reporting items for systematic review and meta-analysis
MeSH: Medical subject headings
PICOS: Population, intervention, comparator, outcome, and study design
PEDro: Physiotherapy evidence database
GOSH: Graphical display of study heterogeneity
R: Correlation coefficient
SMD: Standardized mean difference
LRT: Likelihood ratio tests

## Footnotes

1 The beta coefficients represent the estimated change in the effect size for each unit change in the predictor variable, while holding all other variables constant. For example, for the mean duration of single stretching exercise the unit is minutes. Therefore, the beta represents the estimated effect size changes per minute duration of single stretching exercise. The same principle applies for the other variables with their respective units.
